# Avoidance Measures for Patients with Allergic Rhinitis: A Scoping Review

**DOI:** 10.3390/children10020300

**Published:** 2023-02-03

**Authors:** Miguel Tomé, Olga Lourenço

**Affiliations:** 1 FCS-UBI, Faculty of Health Sciences, University of Beira Interior, 6200-506 Covilhã, Portugal; 2 CICS-UBI, Health Sciences Research Centre, University of Beira Interior, 6200-506 Covilhã, Portugal

**Keywords:** allergic rhinitis, hay fever, allergen avoidance, management, quality of life

## Abstract

Environmental allergen control is recommended as an essential part of allergic rhinitis (AR) management guidelines. In this scoping review, our objective is to identify measures of allergen avoidance and to evaluate their effectiveness in the management of AR. We conducted systematic searches for randomized controlled trials and observational studies in PubMed, the Cochrane Central Register of Controlled Trials, and the Web of Science databases. We included all types of control measures based on allergen eviction or reduction in exposure. Overall, 18 studies satisfied our criteria and were thus included for further analysis. The majority of the studies (15 out of 18) reported decreases in overall AR symptom scores, improvements in quality of life, or reductions in medication usage. However, due to the low number of participants and the limitations in study designs, it is not possible to make a definitive recommendation on the use of these interventions in the management of AR. A multifaceted approach, including treatment, prevention, and eradication of allergens from the environment, may be necessary to effectively reduce symptoms.

## 1. Introduction

Allergic rhinitis (AR) is a chronic respiratory disease caused by a symptomatic IgE-driven inflammation of the nasal mucosa, that leads to symptoms such as rhinorrhea, nasal congestion, pruritus, and sneezing [1,2]. AR is a very common disease, affecting all ages and socioeconomic backgrounds, with a higher incidence in Western countries [3] and a global prevalence estimated between 10 and 40% [2,4]. Many patients with AR develop symptoms before adulthood, with nearly half of such patients becoming symptomatic during childhood. Young children, aged 6 to 7, have a reported global prevalence greater than 8.5%, and in adolescents aged 13 to 14, reported global prevalence has been reported to be greater than 14% [5]. Despite its prevalence, AR is still an underdiagnosed, undertreated, and many times ignored condition [1,2]. Not only does it affect the general health and quality of life (QoL) of patients, but also has a negative impact on school and work productivity [3,4]. Therefore, the correct management of AR is beneficial for both the patients and society as a whole.

The main goals of treatment include restoring the normal function of the upper airways and improving QoL through patient education, allergen avoidance/control measures, pharmacotherapy, and in some cases, allergen-specific immunotherapy [1,2,3,4]. 

AR occurs through host sensitization to foreign proteins—the allergens—across the nasal mucosal barrier via dendritic cells and naive CD4-positive lymphocytes, with the generation of antigen-specific type 2 lymphocytes and IgE-secreting plasma cells. Allergen sensitization patterns vary significantly by geography, genetics, living conditions, and climate, amongst other factors [6,7,8]. Moreover, different types of allergens such as pollen, house dust mites (HDM), and pet dander can cause AR [9,10]. 

Regardless, subsequent allergen challenge across the nasal mucosa of the sensitized individuals results in cross-linking of the IgE bound to the surface of mast cells with degranulation, mediator, chemokine, and cytokine synthesis and secretion, leading to the recruitment of other inflammatory cells. It is crucial to distinguish between sensitization to aero-allergens and AR. Around 60% of the total amount of sensitizations to aero-allergens are clinically relevant and associated with patient-reported clinical symptoms, the remaining cases are irrelevant [11]. This distinction is particularly important when considering immunotherapy [12]. 

Children often struggle with medication compliance, and some parents hesitate to give medication, namely corticosteroids, at a young age. Allergen avoidance measures could then be a good option for parents to apply to their offspring, to manage AR symptoms. 

Although allergen avoidance measures can play an important part in the management of AR, there is scarce information on the different measures that can be applied, their validity from a scientific perspective, and most importantly, if they can have a beneficial effect. 

In this scoping review, our objective is to identify measures of allergen avoidance and to evaluate their effectiveness. The impact of these measures was evaluated mainly from the following four perspectives: disease control, nasal symptoms score, medication usage, and QoL. 

## 2. Materials and Methods

This scoping review followed the guidance developed by members of the Joanna Briggs Institute [13]. The authors conducted searches for randomized control trials (RCT) and observational studies in PubMed, the Cochrane Central Register of Controlled Trials, and the Web of Science databases. These databases were examined from inception until the 31st of May 2022. We included papers published in English, Portuguese, French, or Spanish. 

All individuals with a clinical diagnosis of AR were included; there was no age or sex restriction. We included all types of control measures based on allergen eviction or reduction in exposure, for instance, but not limited to the use of acaricides, use of impermeable covers for mattress/pillows, cigarette smoke eviction, air pollutants eviction, use of air filters (HEPA), and combinations of different interventions. Exclusion criteria included: (a) papers without translation to one of the languages in the inclusion criteria; (b) studies with incomplete outcome data. 

The search strategy applied for the PubMed database served as a model for the remainder databases; the following expression was used: (“allergic rhinitis” OR “hay fever” OR pollinosis) AND (avoidance OR “secondary prevention” OR nonpharmacological) AND (allergen* OR pollen OR “dust mite*” OR “air filter*” OR “air pollution”). 

Additionally, we retrieved relevant systematic reviews and scanned their reference lists for additional trials.

Both authors checked titles and abstracts identified from the searches independently. Afterward, the full texts of all studies of possible relevance for assessment were obtained. Reference lists of the identified papers were scanned for further relevant articles. The authors decided which trials satisfied the inclusion criteria and graded their methodological quality. The two authors using a standardized form extracted the data independently. All discrepancies were resolved with discussion among authors. Data extraction included article reference (first author, title, and date), study design, population (number of individuals, sex, age, type of intervention), and outcome(s). 

## 3. Results

**Figure 1:**
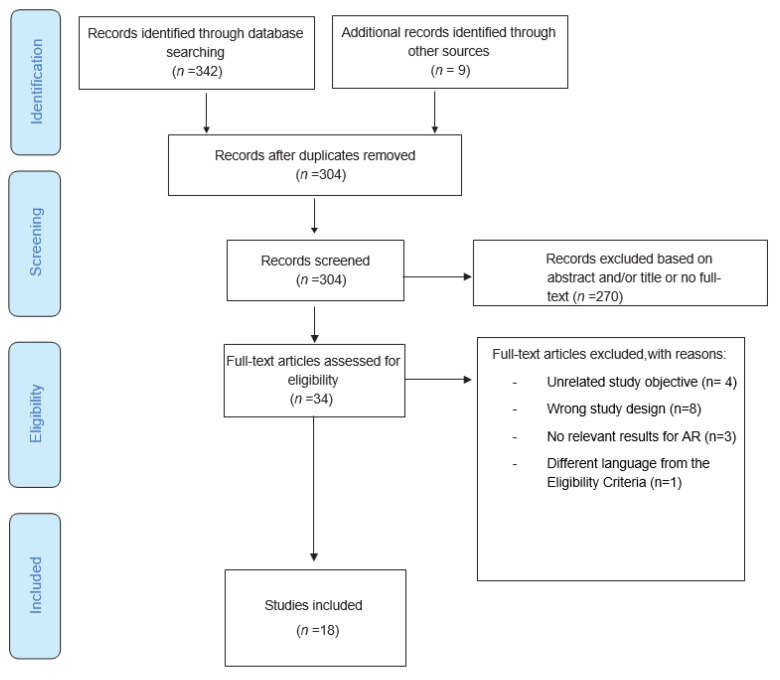
Flowchart of the selection process.

The search process yielded the identification of 342 possible articles in the three databases (Pubmed, *n* = 43; Cochrane Central Register of Controlled Trials, *n* = 59; Web of Science, *n* = 240). Nine additional studies were identified through the reference lists. Afterward, 47 duplicated articles were removed, and the remaining articles were screened based on titles, abstracts, and accessibility to full text, which resulted in the exclusion of 270 papers. From the 34 papers eligible for full-text reading, 18 studies were included in this scoping review. Figure 1 shows the flowchart of the selection process.

Of the selected articles, 7 were randomized control trials [14,15,16,17,18,19,20], 8 were crossover studies [21,22,23,24,25,26,27,28], 1 was a parallel trial [29], 1 was an observational study [30], and 1 was a randomized placebo-controlled 2 × 2 factorial trial [31]. The studies analyzed had different outcome measures and the measures were tested considering different allergens. House dust mite (HDM) was the most common allergen sensitization and most measures were designed to control HDM exposure [14,15,17,21,23,24,26,27,28,29,30,31]. Out of the 18 studies, 5 of them had participants with ages ranging from 6 to 16 years old [17,18,24,29,30]. The general characteristics and findings of these studies are presented in Table 1. Due to substantial methodological differences, meta-analysis was not conducted in this review.

### 3.1. Environmental Control Measures

Four studies [16,25,27,28] evaluated the impact of air filtration systems. In the study by Antonicelli et al. [28], nine patients with AR (10 to 28 years of age), sensitized to HDM used a filtration unit equipped with HEPA filters for 8 weeks. Similar filtration units were used for 4 weeks in another study [27], that included 32 patients with perennial allergic rhinitis (PAR) and/or asthma (6 to 61 year of age) sensitized to HDM. There was a reduction in HDM allergen levels shown in one study [27], but with no significant differences in the other [28]. Moreover, the study by Antonicelli [28], also failed to have any significant impact on rhinitis and asthma symptom scores.

A bigger study, by Li et al. [16], also studied the impact of an air purifier on allergy symptom scores and QoL. This study included 90 patients with AR (aged 18 to 65 years) sensitized to Artemisia pollen. No differences were observed in the Rhinoconjunctivitis Quality of Life Questionnaire (RQLQ) and tolerability to the air purifier decreased progressively each week [16].

A different kind of system (fresh air ventilator equipped with pollen filters) was tested by Brehler et al. [25] in a total of 44 adult patients with seasonal AR, allergic conjunctivitis, and asthma. In summary, three of the studies ( showed improvement in allergy symptom scores and QoL; two showed a reduction in medication usage [25,27], and one reported an increase in morning peak expiratory flow rate (PEFR) for patients with AR [25].

Two studies [18,30] evaluated temperature and/or humidity control on HDM allergen levels. Manuyakorn et al. [30] enrolled 7 children with persistent AR, sensitized to HDM and evaluated the use of a temperature and humidity control machine. The authors report a significant reduction in the Total Nasal Symptom Score (TNSS) in the 2nd and 4th months. Moreover, 70% of the patients were able to stop using their intranasal corticosteroids by the end of the study. Noteworthy, the HDM concentration was significantly reduced only after four months into the study.

The study by Mohan et al. [18] investigated the effects of temperature control by employing a temperature-controlled laminar airflow. Fifty-two children with allergic asthma and rhinitis took part in this study. The intervention was not associated with a statistically significant improvement in parent-reported sleep disturbance compared with placebo after 1 year of treatment, nor in self-reported sleep disturbance. Aired assessments using wristwatch actigraphy were made in 15 individuals and showed statistically non-significant improvements in sleep quality.

Three studies [15,23,29] intervened on bed covers and/or mattresses, investigating the effectiveness of different approaches in reducing allergen exposure while sleeping. The study by Terreehorst et al. [15] included 232 patients with AR and/or asthma (8 to 50 years of age), and evaluated the use of bed covers impermeable to mite allergens. These covers reduced HDM concentration in the mattresses of the intervention group. However, no significant difference in VAS, nasal allergen-provocation tests, or daily symptom score was observed. The study by Berings et al. [23] evaluated the use of probiotics-impregnated bed covers in 24 adult patients with AR sensitized to HDM. Unexpectedly HDM concentrations were reduced both in the control and in the intervention groups. The latter showed significant overall symptom improvement compared to baseline and improvement in the QoL scores. Minor changes in medication intake were also observed, but those were not significant. The study by Jeon et al., published in 2019 [29] evaluated the daily vacuuming of mattresses. Forty children with mild PAR, monosensitized to HDM were included. All symptom scores decreased significantly after 2 weeks as well as total symptom scores, sneezing, rhinorrhea, nasal obstruction, and itching in the experimental group. The collected dust weight was significantly decreased by week 2 in the active group, although the concentrations of HDM did not change.

Two studies [14,24] evaluated the use of acaricides on indoor HDM allergen levels. The study by Chen et al. [24], included 66 children (3 to 12 years of age) with AR and asthma, sensitized to HDM. HDM allergen levels were lower in the intervention group when compared to the control group. In this study, Visual Analog Scale (VAS) score, the Rhinitis Control Assessment Test (RCAT) score, and the RQLQ score significantly improved in the group using the acaricide. In the study by Kniest et al. [14], 20 patients with PAR (12 to 36 years old), sensitized to HDM, divided equally between the intervention and the control group were included. The protocol of acaridical cleaning led to a decrease in daily symptoms and total IgE in the intervention group. No irritation or toxic effects were associated with the use of the acaricides.

### 3.2. Individual Control Measures

Two studies [19,22] evaluated the use of nasal filters when the patients were outdoors in a park during pollen season. Kenney et al. [22] included 65 adult patients with AR sensitized to grass pollen. The other study, by O’Meara et al. [19], included 46 adult patients sensitized to mixed ragweed. Both studies showed an improvement in symptom scores for the intervention group. In the study by Kenney et al. [22], differences in the daily Total Ocular Symptom Score (TOSS) and the Forced Expiratory Volume in the first second (FEV1) were insignificant. In terms of usability and tolerability of the device, global discomfort was considered mild.

Two studies [20,26] evaluated the use of nasal topical microemulsions. In Andersson et al., 20 adult patients with PAR sensitized to HDM applied the nasal topical microemulsion (a mixture of glycerol-monooleate, propylene-glycol, polyethylene-glycol 400, sesame oil, and polysorbate-80 in isotonic saline) or placebo. The study showed a reduction in nasal symptoms on days 3, 4, and 6 of treatment [26]. In a multicenter, multinational study by Ojeda et al. [20], 110 patients with moderate to severe AR or rhinoconjunctivitis sensitized to grass, birch, or olive tree pollens applied a topical microemulsion or placebo. Allergic symptoms were lower in the intervention group along with lower global scores in the mini-RQLQ, indicating a better overall QoL. However, the differences between groups were not statistically significant. Overall, the topical microemulsions were considered safe and well tolerated, and no major discomfort was reported.

### 3.3. Combined Measures

Three [17,21,31] studies applied a combination of different measures. The study by Stillerman et al., included the use of HEPA filters and pillow encasements [21]. Thirty-five adults with Perennial Allergic RhinoConjunctivitis (PARC) sensitized to HDM, dog, or cat were included. In this study, the reduction in the allergen-sized particulate matter led to significant improvements in nocturnal nasal and ocular allergy symptoms and the QoL score. In a Korean study by Moon et al., 30 adult patients with AR sensitized to HDM were evaluated. The study included several combined measures: bed covers, vinyl mattress covers, daily wet cleaning of the floor, fortnightly boil washing of top bedding cover, and removal of soft furnishings. The HDM concentration was reduced and symptom scores improved showing a significant benefit of the proposed measures [17]. An Italian study by Incorvaia included 25 patients with persistent AR sensitized to HDM, divided into four groups with different combinations of bed casings and acaricide [31]. Although there was a significant improvement when compared with the baseline, there was no difference between the intervention and control groups. 

## 4. Discussion

As was pointed out in the Introduction, allergen eviction is one of the pillars in the management of allergic diseases. Despite this, the literature involving allergen avoidance in patients with AR is scarce, making it difficult to recommend environmental modifications or measures to reduce allergen exposure. In a 2008 systematic review by Gøtzsche et al. [32] that assessed the effects of reducing exposure to house dust mite antigens with environmental measures in patients with asthma, no statistically significant differences were found in asthma symptom scores or medication usage. This systematic review came after several RCTs produced conflicting results regarding the effectiveness of environmental measures. It remains to be established if the same can be concluded regarding AR.

Concerning patients with atopic dermatitis (AD), allergen avoidance measures tackling HDM (considered to be the most relevant allergen in AD), seem to be insufficient for symptom control, although in combination with pharmacological treatment they showed to be beneficial [33]. This seems particularly important since allergen-specific immunotherapy studies in patients with AD showed conflicting results [34].

A Cochrane systematic review of published RCT of mite avoidance measures for perennial AR published in 2001 reported no beneficial effect of physical or chemical interventions, concluding that there is little evidence that a reduction in mite exposure will lead to a sustained improvement in patient’s outcomes. A follow-up of this review published in 2012 also failed to offer any definitive recommendations [35,36]. 

Our main aim with this review is to provide the scope of the strategies of allergen avoidance and control and gather evidence that could endorse recommendations made by healthcare professionals.

The proposed strategies followed two different directions: one focusing on the surrounding environment (for instance the use of acaricides or air filters) and the other focusing on the individual (for instance the use of nasal filters or topical microemulsions) [19,20,22,26]. Although the included volunteers with AR were sensitized to different triggering allergens, most of the studies included interventions that revolved around HDM, and none was intended to reduce mold allergens. Reduction in mites and mite allergens can be attempted in several ways: encasing of mattresses, duvets, and pillows; chemical methods, such as acaricides; and/or control of temperature and humidity in the home. What emerges from these studies is the fact that avoidance measures used alone do not seem to be sufficient. Studies that combined multiple allergen measures [17,21,31] showed more significant results in contrast with other studies that evaluated only one type of measure. This suggests that different ways of handling the exposure or concentration of the allergens could be a more effective way of AR control. 

Nevertheless, an important aspect is that many environmental control measures are costly and cumbersome, which further complicates their applicability outside a clinical trial.

In the case of exposure to airborne pollens, most environmental control measures would be inefficient, as they imply that the patient stays indoors with closed windows. In such a setting, individual measures based on the use of filters or nasal microemulsions might be an interesting option. However, both studies on the use of nasal filters [19,22] were very limited in time, failing to demonstrate the usability of such filters during several days or even weeks, as in a typical pollen season. Three mostly overlooked aspects to take into account when recommending such devices are that the filter should fit appropriately in the nostrils, efficiently providing a barrier to the air; should be discreet and allow for an active lifestyle. The alternative use of microemulsions [20,26] seems promising, as this is a less invasive approach, although no significant differences in symptoms scores were obtained to date.

During the COVID-19 pandemic, measures such as the use of facemasks and greater ventilation of homes and workspaces showed some impact on AR symptoms [37,38]. In theory, these observations argue in favor of a combination of environmental and individual measures.

Overall, although the majority of studies showed improvements in at least one outcome, the studies presented some limitations. Excluding one study [15], the remainder had a limited number of participants, which makes extrapolation of the findings to the general population with AR difficult. Some studies [27,28] showed contradictory results when studying the same avoidance measure applied to the same allergen—in this case, HDM. This hinders any conclusion for or against the proposed measure. The majority of studies only dealt with monosensitization, mainly to HDM, and allergens such as molds were not the subject of any study. Approaches must be tailored to the individual’s sensitization profile in that most individuals with AR are sensitized to multiple indoor and outdoor allergens. Any approach that does not achieve adequate exposure reduction to all the relevant allergens is unlikely to show benefits.

Other potential sources of bias should be considered: randomization methods were rarely described; some studies were not truly randomized, or the allocation was not adequately concealed. The reporting of the data was often poor, with authors only reporting that there were or there were no significant differences between the intervention and the control groups.

One very relevant aspect is the fact that some studies included patients taking medication, making it hard to ascertain if the interventions tested were the main reason for the beneficial results obtained. Nevertheless, several studies where patients took no medication did show improvements in the outcomes evaluated [16,17,19,21,22,26,29]. 

Based on these findings, further well-designed studies including a larger number of patients with different allergen sensitivities should be conducted to support the recommendation of these measures.

## 5. Conclusions

The contact of the allergen with the nasal mucosa is needed for the development of symptoms in AR. Intuitively, it follows that exposure avoidance to sensitizing allergens should be beneficial. Nevertheless, recommendations for environmental control measures have been questioned in terms of efficacy. Although allergen avoidance is widely recommended as part of secondary and tertiary prevention strategies for AR, a demonstration of its effectiveness is lacking.

In recent years, several studies and reviews—some performed within the Cochrane collaboration—have evaluated the effectiveness of allergen avoidance for the prevention and control of AR symptoms. The authors of these studies concluded that most preventive measures were able to reduce the level of allergen exposure, and some were better than placebo (or no measure) in reducing the number or severity of episodes of AR as well as affecting positively the overall QoL. Studies involving children as participants have shown that the control of the surrounding environment can have a beneficial impact on the management of the disease, either at a young age or in adolescence [17,24,29,30].

However, very few allergen-avoidance measures have been subjected to appropriate controlled trials, leading to the low quality of the evidence on their behalf. Moreover, it is important to note that complete avoidance of allergens is not always possible, as even small doses of allergens can elicit reactions in susceptible individuals. This makes it difficult to obtain conclusive or statistically significant data when studying symptom prevention. It is also noteworthy that early childhood exposure to microbial antigens may lower the incidence of allergic diseases. This highlights the importance of considering the immune status of the mother, diet, and gut microbiome, as they all have significance in susceptibility to allergies. Therefore, it is necessary to consider a multi-faceted approach that includes a combination of prevention, treatment, and elimination of allergens from the environment to lower the incidence of allergy symptoms. Sublingual or subdermal antigen-specific desensitization is also an effective approach in certain situations. By taking a comprehensive and holistic approach, we can hope to improve outcomes for individuals with allergies.

## Figures and Tables

**Table 1:** Main characteristics and findings of the included studies.

Author, Year	Study Design	Patient Group (n; Age (mean = ±std))	Intervention(s)	Outcomes Evaluated	Results
Andersson M, 2011 [26]	Randomized, double-blinded, crossover trial	20 patients with PAR sensitized to HDM Age = (19–48)	Nasal topical microemulsion	Nasal symptoms score (four-point scale) TNSS	. reduction in nasal symptoms with the microemulsion from baseline . reduction in symptoms when comparing microemulsion and placebo on treatment days 3, 4, and 6
Antonicelli L, 1991 [28]	Randomized cross-over study	9 patients with AR sensitized to HDM Age = 16 yrs (std not given)	HEPA filter	HDM (*Der p1*, *Der f1* and *Der m1*) allergen level rhinitis and asthma symptom scores	. no significant difference in floor allergen levels with HEPA filter use . no difference in rhinitis symptom scores
Berings M, 2017 [23]	Randomized, double-blind, placebo-controlled, crossover trial	24 patients with AR sensitized to HDM Age = not given.	Probiotics-impregnated bed covers	*Der p1* levels in mattress and pillow dust samples Symptoms score (VAS) QoL (RQLQ and NRQLQ) Medication use	. no difference in *Der p1* levels between groups . significant improvement compared to the baseline of symptoms and QoL scores in the intervention group . no significant changes in medication use
Brehler R, 2003 [25]	Placebo-controlled, double-blind, crossover study	44 patients with seasonal AR, allergic conjunctivitis, and asthma Age = not given	Active filter was used	Symptom score PEFR Medication use ECP levels	. reductions in symptoms, use of medication, and increase in PEFR were bigger in volunteers with exclusively hay fever. . no effect on ECP levels
Chen M, 2021 [24]	Randomized, double-blind, placebo-controlled, crossover study	50 patients with AR and asthma sensitized to HDM Age = 6 (std not given)	Acaricidal bait	Symptoms score (VAS) AR control (RCAT) Asthma control (ACQ-5) QoL (RQLQ) HDM allergen levels	. significant improvement in VAS, RCAT, and RQLQ scores in the acaricidal bait . no significant improvement in ACQ-5 . HDM allergen levels significantly decreased
Incorvaia C, 2008 [31]	Randomized placebo-controlled 2 x 2 factorial trial	25 patients with persistent AR sensitized to HDM IG: 12 CG: 13 Age = not given.	Bed casings + acaricide	QoL (RQLQ)	. significant improvement in RQLQ compared to the baseline . no difference when compared to the placebo
Jeon YH, 2019 [29]	Single-blind, randomized parallel trial	40 patients with mild PAR sensitized only to HDM IG: 20; 8.90 ± 2.2 yrs CG: 20; 8.45 ± 2.2 yrs	Daily vacuuming of mattresses	Symptoms score (VAS) dust weight and concentration of *Der p1* and *Der f1*	. significant improvement in all symptom scores after 2 weeks in the intervention group . reduction in the collected dust weight . no significant change in Der p1 and Der f1 concentrations
Kenney P, 2015 [22]	Randomized, double-blind, placebo-controlled crossover clinical trial	65 AR patients sensitized to grass pollen Age = 24.8 ± 6.1 yrs	Nasal filters	TNSS TOSS FEV1	. significant reduction in daily TNSS and maximum TNSS compared with placebo . no difference in daily TOSS . no difference in FEV1 between placebo and filter
Kniest F, 1991 [14]	Double-blind matched pair controlled trial	20 patients with PAR sensitized to HDM IG: 10; 19 yrs (std not given) CG: 10; 21.5 yrs (std not given)	intensive home cleaning with or without acaricide (solidified benzyl benzoate)	Daily symptom score Medication scores Physician assessment Total and mite-specific serum IgE Nose eosinophils Guanine exposure	. reduction in daily symptoms and total IgE with the use of acaricide . no effects with intensive cleaning ‘only’
Li L, 2020 [16]	Randomized, double-blind, controlled trial	90 AR patients sensitized to Artemisia pollen IG: 45; 35.5 ± 8,2 yrs CG: 45; 36.1 ± 9,2 yrs	HEPA filter	Symptom severity (VAS) QoL (VAS) RQLQ Epworth Sleepiness Scale	. rhinitis symptom scores showed significant differences between IG and CG . no differences in RQLQ between groups . nasal symptom score, allergy symptom score (VAS), Epworth Sleepiness Scale score decreased progressively each week
Manuyakorn W, 2015 [30]	Observational study	7 Patients with persistent AR who were skin prick test positive for HDM Age = 9.8 ± 1,7 yrs.	Use of a temperature and humidity control machine	TNSS and the level of dust mite allergens (Der p1 and Der f1)	There was a significant reduction in TNSS at 2 and 4 months and 70% of the patients were able to stop using their intranasal corticosteroids by the end of the experiment. There was a notable reduction in the levels of *Der f1* as early as 2 months after installing the machine but this reduction became significant only after 4 months and Der f 1 level remained low until the end of the experiment.
Mohan L, 2011 [18]	Randomized control-trial	52 patients with allergic asthma and rhinitis sensitized to a perennial allergen IG = 36 CG = 16 Age = 8–16	Nocturnal temperature-controlled laminar airflow (TLA)	Sleep quality (PSQ; CSHQ and wristwatch actigraphy	. no significant improvement in sleep quality
Moon JS, 1999 [17]	Open randomized controlled trial	30 patients with AR sensitized to HDM IG: 15 CG: 15 Age = 15.6 yrs (for total patients)	Bed covers + vinyl mattress cover * daily wet cleaning of floors + fortnight washing of top bedding cover + removal of soft furnishings	Daily rhinitis symptoms scores HDM load	. HDM loads were significantly reduced in the intervention group compared to the control group . significant improvement in symptoms scores in the intervention group
Ojeda P, 2013 [20]	Randomized controlled double-blind clinical trial	110 patients with moderate to severe AR or rhino-conjunctivitis sensitized to grass, birch, or olive tree pollens IG: 55; 32.6 ± 9.9 yrs CG: 55; 34.9 ± 11.5 yrs	Nasal topical microemulsion	QoL (RQLQ) Nasal, ocular, and lung symptoms Medication usage	. reduction in symptoms in the intervention group . improvement in QoL although without statistically significant differences between groups
O’Meara TJ, 2005 [19]	Double-blind placebo-controlled trial	46 patients with AR sensitized to mixed ragweed IG: 22; 51.1 yrs (std not given) CG: 24; 50.3 yrs (std not given)	Nasal filters	Symptoms score (MSC and TSC) Peak nasal inspiratory flow	. MSC scores decreased in the intervention group and increased in the placebo group compared with baseline scores . TSC scores decreased in the intervention group when compared with the placebo group . no difference between groups in peak nasal inspiratory flow
Reisman R, 1990 [27]	Double-blind cross-over randomized controlled trial	32 patients with PAR and/or asthma sensitized to HDM Age = 27.5 yrs (std not given)	HEPA filter	Particulate counts in bedroom air Symptom score Medication score Patient’s subjective response to treatment	. reduction in particulate counts in bedroom air . reduction in symptom and medication scores
Stillerman A et al., 2010 [21]	Randomized, double-blind, placebo-controlled, crossover trial	35 PARC patients sensitized to HDM, dog or cat Age = 39.1 ± 11.9 yrs	HEPA filter + pillow encasement	TSS QoL (NRQLQ)	. significant improvements in TSS and QoL
Terreehorst I, 2003 [15]	Double-blind randomized controlled trial	232 patients with AR and/or asthma IG: 114; 25.7 ± 1.1 yrs CG: 118; 26.9 ± 1.1 yrs	Bed covers	Daily symptom score (VAS) Nasal allergen-provocation test score Der p1 and Der f1 concentration	. concentrations of *Der p1* and *Der f1* in the mattress sample was significantly lower in the impermeable cover group when compared with the control group. . no significant difference between groups on daily symptom score, or nasal allergen provocation test

ACQ-5 = Asthma Control Questionnaire-5; CG = Control Group; CSHQ = Child’s Sleep Habits Questionnaire; Der p = *Dermatophagoides pteronyssinus*; Der f = *Dermatophagoides farinea*; ECP = Eosinophil Cationic Protein; FEV1 = Forced Expiratory Volume in the first second; HDM = House Dust Mite; HEPA = High-Efficiency Particulate Air; IG = Intervention Group; MSC = Major Symptom Complex; NRQLQ = Nocturnal Rhinoconjunctivitis Quality of Life Questionnaire; PAR = Perennial Allergic Rhinitis; PARC = Perennial Allergic RhinoConjunctivitis; PEFR = Peak Expiratory Flow Rate; PSQ = Pediatric Sleep Questionnaire; QoL = Quality of Life; RCAT = Rhinitis Control Assessment Test; RQLQ = Rhinoconjunctivitis Quality of Life Questionnaire; std = standard deviation; TNSS = Total Nasal Symptom Score; TOSS = Total Ocular Symptom Score; TSC = Total Symptom Complex; TSS = Total Symptom Scores VAS = Visual Analogue Scale.

## Data Availability

Not applicable.
